# Trends of vaccine-preventable diseases in Afghanistan from the Disease Early Warning System, 2009–2015

**DOI:** 10.1371/journal.pone.0178677

**Published:** 2017-06-01

**Authors:** Abram L. Wagner, Mohammad Y. Mubarak, Laura E. Johnson, Julia M. Porth, Jenna E. Yousif, Matthew L. Boulton

**Affiliations:** 1 Department of Epidemiology, School of Public Health, University of Michigan, 1415 Washington Heights, Ann Arbor, MI, United States of America; 2 Department of Microbiology, Kabul Medical University, Kabul, Afghanistan; 3 Division of Infectious Diseases, Department of Internal Medicine, University of Michigan, Ann Arbor, University of Michigan Medical School, 1500 East Medical Center Drive, Ann Arbor, MI, United States of America; Public Health England, UNITED KINGDOM

## Abstract

**Background:**

Afghanistan’s public health system was neglected during decades of military and civil conflict, and trends in infectious disease occurrence remain poorly characterized. This study examines cyclical and long-term trends of six vaccine-preventable diseases: pneumonia, diarrhea, meningitis, typhoid, measles, and acute viral hepatitis.

**Methods:**

Using weekly data collected between 2009 and 2015 through Afghanistan’s Disease Early Warning System, we calculated monthly case counts, and fit a Poisson regression with a Fourier transformation for seasonal cycles and dummy variables for year.

**Results:**

We found the greatest incidence of diarrhea and typhoid in the summer, pneumonia in the winter, and measles in the late spring. Meningitis and acute viral hepatitis did not demonstrate substantial seasonality. Rates of pneumonia and diarrhea were constant across years whereas rates of meningitis, typhoid, and acute viral hepatitis decreased. Measles incidence increased in 2015.

**Conclusions:**

Communicable disease reporting systems can guide public health operations–such as the implementation of new vaccines, and permit evaluation of health interventions. For example, measles supplementary immunization activities in Afghanistan have not slowed long-term transmission of the disease, but decreases in typhoid fever and acute viral hepatitis are probably tied to improvements in sanitation in the country.

## Introduction

Afghanistan has suffered for decades under military and civil conflict which, combined with natural disasters, has seriously impaired economic development [1]. These conflicts have led to over a half million internally-displaced persons (IDPs) moving through the country with precarious ties to the health care infrastructure [2]. In addition, although Afghanistan still is majority rural, it is urbanizing at one of the world’s highest annual rates– 6% [3]–and 5 million people may be currently living in slums [4]. Public health infrastructure, including sanitation and vaccination programs, are slowly rebuilding since the overthrow of the Taliban government in 2001, and health care facilities in Afghanistan are recognized for tending to marginalized groups, such as women and the very poor [5]. However, it remains clear that Afghanistan faces many challenges in fighting infectious diseases: sanitation, such as piped water and private latrines, is limited for slum dwellers and IDPs [6]; the nutrition status of children is poor—almost half of children have anemia and vitamin A deficiency [7]; and substantial disparities exist in childhood immunization coverage between cities and rural areas [8].

The Expanded Program on Immunization (EPI) in Afghanistan has included the Bacillus Calmette-Guérin (BCG), the diphtheria-tetanus-pertussis (DTP), polio, and measles vaccines. The hepatitis B vaccine was introduced to the EPI in 2006, the *Haemophilus influenzae* type b (Hib) vaccine in 2009, the 13-valent pneumococcal conjugate vaccine (PCV13) in 2013, and the rotavirus vaccine in 2014 [9]. Other vaccines for hepatitis A [10], hepatitis E [11], or typhoid (including a newer generation of typhoid conjugate vaccines [12]) are currently not on the EPI.

Accurate information on the epidemiology of infectious diseases in Afghanistan is mostly very dated [13], or tied to U.S. military efforts in the region, which limits generalizability to the country’s general population [14–16]. Ikram et al. analyzed disease surveillance systems in Afghanistan through 2011 [17], providing an informative descriptive analyses but no statistical tests to explore trends in occurrence of disease over time. Long-term and seasonal trends in infectious diseases in Afghanistan continue to be poorly understood, but these factors can inform planning for disease control efforts, including vaccination programs and sanitation services.

This study examines cyclical and long-term trends of six diseases that are vaccine preventable: pneumonia, diarrhea, meningitis, typhoid, measles, and acute viral hepatitis. The typhoid vaccine is not currently included in Afghanistan’s EPI, however, and pneumonia, diarrhea, and meningitis can be caused by many pathogens—only some of which are vaccine preventable. Many cases of these diseases could also be prevented following straightforward improvements in nutrition and sanitation services. Understanding the seasonality and burden of these diseases may provide insight into the success of Afghanistan’s EPI, while also providing critical trend data needed to set disease control priorities and focus interventions on improving vaccination coverage and informing efforts to enhance access to sanitation systems.

## Methods

### Dataset

The Disease Early Warning System (DEWS) is a sentinel surveillance system in Afghanistan, which has received funding from the US Agency for International Development and is administered by the Afghanistan government. The sentinel sites are located in all provinces of Afghanistan and include regional/national hospitals, provincial hospitals, district hospitals, comprehensive health centers, basic health centers, and other special hospitals or private clinics. DEWS was launched in 2006 and has expanded rapidly since then; the number of sentinel sites increased from 131 in January 2009 to 503 in December 2015. Not every sentinel site will submit a report each week; in the study period, though, 99.4% of sentinel sites, on average, submitted a weekly report, with this figure ranging from 89.7% to 100%. The central DEWS database is located at the Ministry of Public Health central office in Kabul, while there are regional databases. Within each region, a DEWS staff member process reports from health facilities and submit aggregated reports to the central database. These regional databases are not publicly available. Each week, sentinel sites submit aggregated information about the number of consultations as well as more detailed information about suspected cases of “DEWS-targeted” diseases, which have varied over time, but include pneumonia, diarrhea, meningitis, acute viral hepatitis, measles, (neonatal) tetanus, malaria and typhoid. Outbreak investigations of other diseases, including chickenpox, pertussis, swine flu, hemorrhagic fever, food poisoning, and gas poisoning, are only intermittently included in the reports and not addressed further in this study. The cases reported into DEWS are diagnosed based on clinical criteria, and no information about standardized laboratory testing is included. No individual-level or geographic information is available in the dataset.

This manuscript analyzes weekly reports of six diseases in DEWS that are fully or at least in part vaccine-preventable: pneumonia, diarrhea, meningitis, typhoid, measles, and acute viral hepatitis. There are a few notable absences in this analysis. While the BCG vaccine is covered in the Afghan EPI, the DEWS does not take reports on tuberculosis cases. Though tetanus cases are reported in the DEWS, there was an insufficient number of cases throughout the study period to permit a meaningful analysis.

Due to periodic government service interruptions and funding limitations for public health programs, there were no reports available for the weeks of 9 March 2009 to 11 May 2009, 21 December 2009 to 11 January 2010, 9 August 2010 to 9 January 2012, 19 July 2014, and 12 April 2015. Moreover, additional information has become available over time; for instance, reports of pneumonia and diarrhea deaths were available from the beginning of the study period, but reports of deaths due to measles and acute viral hepatitis were not added until mid-2014 (because few deaths due to typhoid were reported, we have not included an analysis of them); and starting in February 2015, details about diarrheal symptoms (watery, watery with dehydration, bloody) were reported more consistently.

### Monthly case counts

DEWS is reported by week, but for some analyses, we collapsed data into month-specific groups to produce more stable results. Because weeks can cross month boundaries, we assumed an equal number of cases on each day of the week, and divided the number of cases into each month accordingly.

Because the number of sentinel sites reporting data into the surveillance system increased over time, we also used adjusted monthly case counts in some analyses, where the number of cases per sentinel site is multiplied by a standard of 500 (the number of reporting sites by the end of the study period). For example, during the first week of January 2015, there were 393 reporting sites. To obtain the adjusted monthly pneumonia case count, we divided the monthly case count value of 82,200 by 393 and multiplied by 500 for a final value of 104,580.

### Statistical analysis

We fit the adjusted monthly case counts for each disease into a time-series Poisson regression model similar to a previous paper [18]. The model included two harmonic terms for the monthly cycle over a year, and dummy variables for each year:

$$cases=\beta_{0}+\beta_{1}\times \sin(2\pi \times \frac{month}{12})+\beta_{2}\times \cos(2\pi \times \frac{month}{12})+\beta_{3}\times year\;2010+\beta_{4}\times year\;2011+\beta_{5}\times year\;2013+\beta_{6}\times year\;2014+\beta_{7}\times year\;2015$$

This model had a log link and was estimated with robust variance estimation. Because the number of sentinel sites increased over the study period, we set the offset to be the number of sentinel sites reporting cases. Using this model, we were able to compute the average monthly case count each year, adjusting for the effect of season. We also specified another model with year as a continuous variable, in order to test for long-term trends in disease incidence.

Seasonal indices were calculated, in accordance with previous literature [19], by dividing the adjusted monthly case count by the average monthly case count each year. For example, according to the Poisson regression, 55,950 cases of pneumonia were expected each month in 2015 after controlling for the effect of season, therefore the seasonal index for pneumonia in January 2015 is 82,120 / 55,950 = 1.47. The final seasonal index for pneumonia, 1.87, was calculated by taking the average of the annual values between 2009 and 2015. Standard deviations (SD) and the ratio of the highest seasonal index to lowest seasonal index are presented to describe the spread of the statistics.

We also provide descriptive information about the diseases. The Case Fatality Ratio was calculated as the total number of deaths over the entire study period divided by the number of cases over the study period, multiplied by 100,000. The number of cases, and the number of deaths, in children <5 years of age was also available for some diseases over a shorter period of time. Finally, the distribution of diarrhea by symptoms (watery, watery with dehydration, and bloody) was compared across weeks using a Kruskall-Wallis Test. Significance for all tests was set a value of α = 0.05, and all data were analyzed in SAS version 9.4 (SAS Institute, Cary, NC, USA).

Because of missing data, we were unable to calculate the Case Fatality Ratio for typhoid, or the number of deaths <5 years for pneumonia, diarrhea, meningitis, and typhoid.

### Ethical approval

This study was a secondary analysis of publicly-available, aggregated data from a surveillance system with no personally-identifiable information [20]. Therefore, we did not seek ethical review.

## Results

This study includes 358 weeks’ worth of communicable disease reports from DEWS collected between January 12, 2009, and December 20, 2015. The records from this time period encompass a total of 86,138 weekly reports from sentinel sites.

For 2015, the adjusted monthly case count was 55,950 for pneumonia (95% CI: 49,064, 63,800), 179,532 for diarrhea (95% CI: 162,285, 198,632), 789 for meningitis (95% CI: 679, 916), 6,445 for typhoid (95% CI: 5,706, 7,279), 922 for measles (95% CI: 712, 1,195), and 918 for acute viral hepatitis (95% CI: 820, 1,028).

**Fig 1:**
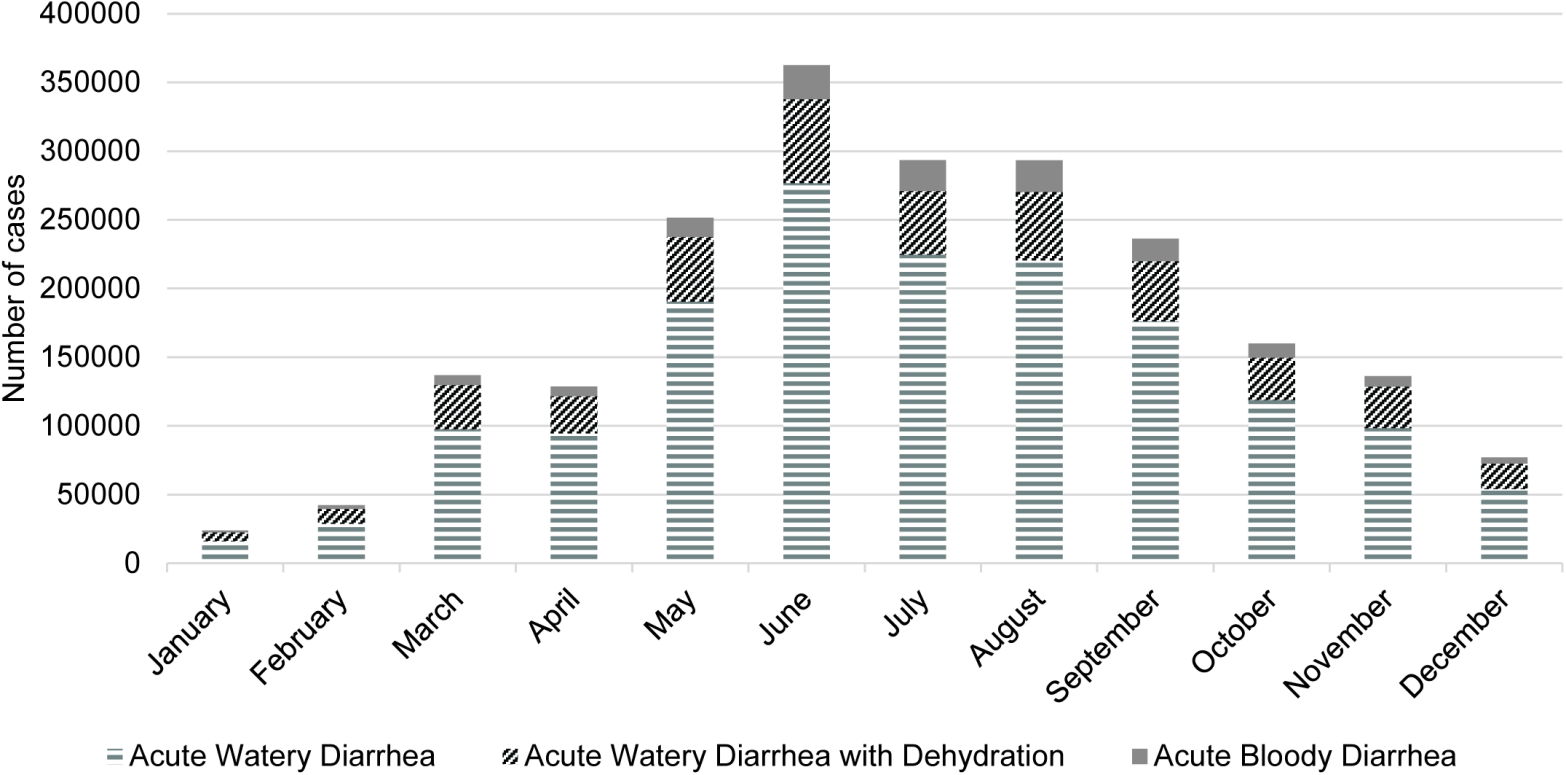
Diarrhea characteristics in Afghanistan’s Disease Early Warning System, 2015.

Meningitis was most often fatal, with 8,817.2 deaths reported per 100,000 cases. In order, the next most fatal diseases were measles (309.1/100,000), pneumonia (319.6/100,000), viral hepatitis (127.6/100,000), and diarrhea (22.9/100,000). Characteristics of the diseases are shown in Table 1. The vast majority of the cases for these diseases occurred in children <5 years of age, except for acute watery diarrhea with dehydration (49% of cases were <5 years), typhoid (17.2% of cases were <5 years), and acute viral hepatitis (25.5% of cases were <5 years. Diarrhea is a multifarious disease, and for the 46 weeks with data available, 73.3% of diarrhea cases were designated acute watery diarrhea, 20.4% acute watery diarrhea with dehydration, and 6.3% acute bloody diarrhea. The proportion of diarrhea with these symptoms also varied by season (Kruskal-Wallis Test p<0.0001). There was a greater proportion of acute watery diarrhea and acute bloody diarrhea in the summer than other seasons, whereas the proportion of acute watery diarrhea with dehydration was greatest in the winter. Fig 1 shows the monthly distribution of these different types of diarrhea in 2015.

**Table 1:** Characteristics of suspected cases of six diseases from Afghanistan’s Disease Early Warning System, 2009–2015.

	Weeks of observations	Mean	Standard Deviation	Minimum	Maximum
Diarrhea symptoms (% of all diarrhea)					
Acute watery diarrhea	46	73.3	3.0	66.6	78.0
Acute watery diarrhea with dehydration	46	20.4	3.6	15.1	27.7
Acute bloody diarrhea	46	6.3	1.0	4.7	8.5
Proportion of cases <5 years (% of all cases)					
Pneumonia	46	66.1	1.8	62.5	70.0
All diarrhea	46	58.1	1.4	55.3	60.9
Acute watery diarrhea	46	60.3	1.4	56.8	62.9
Acute watery diarrhea with dehydration	46	49.0	1.5	45.2	51.3
Acute bloody diarrhea	46	61.2	2.7	57.5	68.4
Meningitis	46	65.9	7.0	30.5	78.5
Typhoid	46	17.2	1.7	13.7	24.0
Measles	72	71.0	8.8	27.3	87.1
Acute Viral Hepatitis	72	25.5	9.2	8.6	58.8
Proportion of deaths <5 years (% of all deaths)					
Measles	37	89.2	21.8	0.0	100.0
Acute Viral Hepatitis	10	25.0	35.4	0.0	100.0

Table 2 shows the long-term trends in the incidence of the six diseases, adjusted for seasonal cycles and the number of sentinel sites. Pneumonia and diarrhea did not deviate in incidence over time. Compared to 2009, the rate of measles was 0.19 times as high in 2013 (95% CI: 0.12, 0.30) and 0.61 times as high in 2014 (95% CI: 0.34, 1.07), but the incidence of disease was similar in 2015 as in 2009. For meningitis, typhoid, and acute viral hepatitis, there were monotonic trends towards lower incidence of disease during the study period (S1 Table).

**Table 2:** Cyclical and long-term trends in the incidence of suspected cases of six diseases from Afghanistan’s Disease Early Warning System, 2009–2015.

	Pneumonia	Diarrhea	Meningitis	Typhoid	Measles	Acute Viral Hepatitis
	RR (95% CI)	RR (95% CI)	RR (95% CI)	RR (95% CI)	RR (95% CI)	RR (95% CI)
Intercept	111.20 (96.17, 128.57)	322.85 (287.87, 362.06)	3.31 (2.85, 3.86)	30.38 (27.18, 33.96)	2.09 (1.46, 2.99)	2.99 (2.63, 3.40)
sin (2π*month/12)	1.36 (1.25, 1.49)	0.68 (0.63, 0.73)	1.01 (0.92, 1.11)	0.83 (0.77, 0.90)	1.55 (1.31, 1.84)	0.95 (0.88, 1.02)
cos (2π*month/12)	1.45 (1.31, 1.60)	0.56 (0.51, 0.60)	0.89 (0.83, 0.96)	0.68 (0.63, 0.73)	0.59 (0.46, 0.74)	1.00 (0.90, 1.10)
Year						
2009	ref	ref	ref	ref	ref	ref
2010	1.15 (0.96, 1.39)	1.09 (0.93, 1.28)	0.91 (0.76, 1.09)	0.89 (0.77, 1.03)	1.32 (0.91, 1.93)	0.91 (0.75, 1.09)
2011	1.14 (0.96, 1.35)	1.08 (0.94, 1.24)	0.64 (0.54, 0.76)	0.64 (0.55, 0.74)	1.19 (0.80, 1.78)	0.87 (0.72, 1.04)
2013	1.14 (0.94, 1.39)	1.13 (0.97, 1.32)	0.48 (0.40, 0.58)	0.62 (0.54, 0.72)	0.19 (0.12, 0.30)	0.70 (0.59, 0.82)
2014	1.21 (0.93, 1.58)	1.01 (0.85, 1.19)	0.46 (0.35, 0.60)	0.45 (0.36, 0.57)	0.61 (0.34, 1.07)	0.65 (0.51, 0.83)
2015	1.01 (0.83, 1.23)	1.11 (0.96, 1.28)	0.47 (0.39, 0.58)	0.42 (0.36, 0.50)	0.87 (0.56, 1.33)	0.61 (0.52, 0.73)

RR, rate ratio; CI, confidence interval

Results from a Poisson regression with robust variance estimation, which specified an offset of the number of sentinel sites reporting cases.

**Fig 2:**
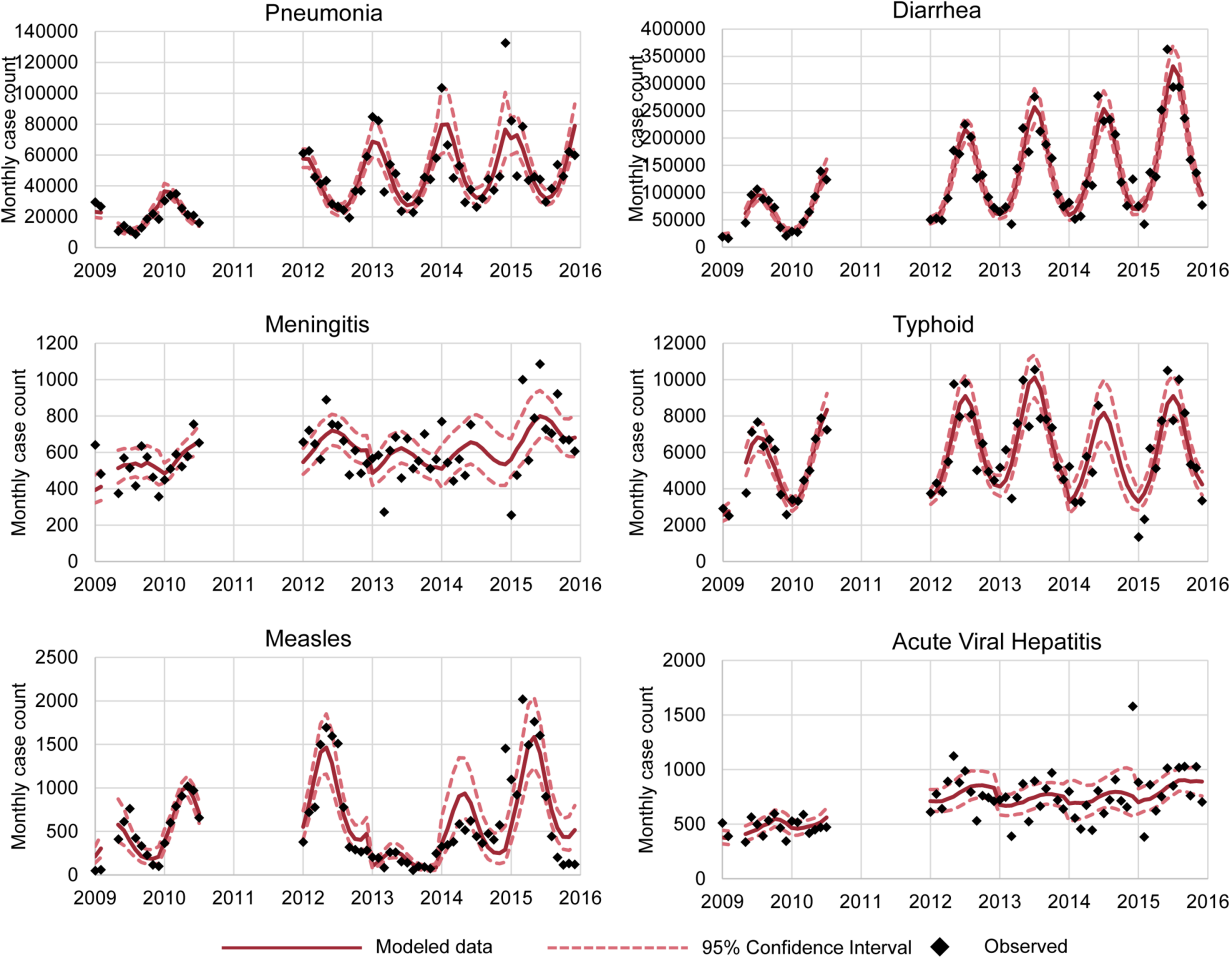
Observed and modeled data from six diseases in Afghanistan’s Disease Early Warning System, 2009–2015.

Several of the diseases demonstrated distinct seasonal trends (Fig 2). The occurrence of diarrhea was 4.69 times greater in the highest incidence month (June) than the lowest incidence month (February). Measles, pneumonia, and typhoid also had notable seasonal swings in disease incidence, with occurrence of disease at least 2 times greater in the highest incidence month than the lowest. Diarrhea and typhoid had the greatest incidence in summer months (in June), whereas pneumonia peaked in winter (in January) and measles in late spring (in May) (Table 3). Meningitis and acute viral hepatitis did not have a large seasonal association in incidence of disease, as shown.

**Table 3:** Annual and seasonal trends for suspected cases of six diseases from Afghanistan’s Disease Early Warning System, 2009–2015.

	Pneumonia	Diarrhea	Meningitis	Typhoid	Measles	Acute Viral Hepatitis
Adjusted monthly case counts^a^ (95% confidence interval)						
2009	55,525 (47,994, 64,245)	162,090 (144,511, 181,808)	1,666 (1,427, 1,945)	15,186 (13,554, 17,016)	1,066 (745, 1,524)	1,493 (1,311, 1,701)
2010	64,070 (57,820, 71,000)	176,364 (157,457, 197,542)	1,509 (1,390, 1,638)	13,481 (12,479, 14,563)	1,409 (1,221, 1,625)	1,353 (1,201, 1,524)
2011	63,275 (58,035, 68,990)	174,854 (160,045, 191,034)	1,066 (969, 1,172)	9,653 (8,753, 1646)	1,272 (1,066, 1,519)	1,292 (1,140, 1,465)
2013	63,580 (55,755, 72,505)	183,470 (163,817, 205,460)	801 (711, 903)	9,470 (8,488, 10,567)	202 (152, 270)	1,039 (927, 1,163)
2014	67,440 (55,055, 82,615)	163,164 (142,915, 186,280)	769 (625, 946)	6,888 (5,662, 8,381)	646 (403, 1,035)	969 (787, 1,195)
2015	55,950 (49,064, 63,800)	179,532 (162,285, 198,632)	789 (679, 916)	6,445 (5,706, 7,279)	922 (712, 1,195)	918 (820, 1,028)
Seasonal indices^b^						
January	1.87	0.53	1.03	0.71	0.92	1.16
February	1.51	0.42	0.96	0.67	0.95	0.88
March	1.22	0.61	0.96	0.81	1.38	0.87
April	1.06	0.97	0.95	1.05	1.66	0.89
May	0.84	1.32	1.00	1.21	1.68	0.89
June	0.78	1.99	1.22	1.54	1.61	1.05
July	0.63	1.95	1.09	1.50	1.31	1.01
August	0.59	1.75	0.89	1.40	0.73	0.98
September	0.82	1.54	1.14	1.30	0.71	1.16
October	0.89	1.11	1.04	1.05	0.54	1.07
November	1.03	0.69	0.86	0.76	0.52	0.97
December	1.46	0.59	0.79	0.56	1.24	1.10
Standard deviation across months	0.39	0.58	0.12	0.34	0.43	0.11
Ratio of highest to lowest month	3.16	4.69	1.54	2.75	3.20	1.34

^a^ Adjusted to 500 sentinel sites reporting cases.

^b^ The highest value for each disease is highlighted in red, and the lowest value is highlighted in green.

## Discussion

A key element of the World Health Organization’s Sustainable Development Goal 3 is the charge to countries to “combat hepatitis, water-borne diseases and other communicable diseases.” This objective is only realistically feasible in the context of a country possessing functional sanitation systems and effective immunization programs. Both have proved challenging for a country like Afghanistan, which has been fraught with almost continuous natural disasters and civil crises over several decades. Many of these same challenges negatively impact other low- and middle-income countries that experience difficulty in controlling diseases even when calling for what are often relatively inexpensive, readily available, and well-established preventive measures. Despite Afghanistan’s difficulties with disease control efforts, we found reasonably steady rates of pneumonia and diarrhea between 2009 and 2015, and a trend towards decreasing rates for meningitis, typhoid, and acute viral hepatitis over the same time period.

Diarrhea, which is a leading cause of infectious death of children globally [21], had the highest adjusted monthly case count of all the diseases analyzed. Introduction of the rotavirus vaccine occurred within the year prior to the end of this study, so there would not have been adequate time for the effects of that introduction to be assessed. Diarrhea in the summer months is a highly prevalent problem for people, especially the burgeoning urban populations who live in crowded slums or in camps for IDPs [6]. The seasonal patterns found in this study show some similarities and differences to findings in nearby countries. For example, Platts-Mills et al. examined seasonality of diarrhea in 8 sites throughout the world [22]. In contrast to our study, the diarrhea high season in Bhaktapur, Nepal, was in early spring; somewhat similar to our study in Afghanistan, they found a diarrhea high season in the summer from July through September in Vellore, India. They found that *Shigella*, enterotoxigenic *E*. *coli*, and *Cryptosporidium* were driving high incidence of diarrhea in the late summer in Vellore, whereas rotavirus was more prominent in the late winter [22]. That the number of cases of diarrhea peaked in the summer provides insight into the most prominent diarrhea-causing pathogens circulating in Afghanistan, namely that bacterial diarrhea, which tends to peak in the summer, may be of more importance in Afghanistan than strains of viral diarrhea, which tend to peak in the winter [23]. The degree to which rotavirus vaccine can impact diarrhea incidence is related to the proportion of diarrhea actually caused by rotavirus. This impact would be increasingly lower if more cases are of bacterial or non-rotavirus viral origin. The etiology of diarrhea in Afghanistan is not well understood; a study of nonbacterial diarrhea in Kabul did find that over three-quarters of the cases had rotavirus [24], although the overall proportion of diarrhea due to nonbacterial causes was not mentioned and the study sites were a convenience sample within one city in Afghanistan, whose populace is still predominantly rural. Further research should investigate the etiology of diarrhea in Afghanistan and changes in diarrhea incidence once the rotavirus vaccine has been more fully implemented.

The majority of the reported cases of acute viral hepatitis were in adults. It is unclear, however, if adults actually suffered more cases of acute viral hepatitis or if children, who tend to have less severe disease presentation and are less likely to be overtly jaundiced, did not have access to care or were misdiagnosed. Historically, incidence of hepatitis A and hepatitis E has been high in Afghanistan and other south-central Asian countries due to the lack of infrastructure maintenance [13]. Improvements in sanitation and vaccine development could explain the declining rates of acute viral hepatitis throughout the study period. We did not find a seasonal trend in viral hepatitis cases. Similar to our study of Afghanistan, a China study found relatively little seasonal variation for hepatitis A or E [19].

Pneumonia cases peaked in winter, as typical for respiratory diseases. While we did not find any reductions in case count by year, the Hib and PCV13 vaccines have only recently been added to the EPI and, like the rotavirus vaccine, probably have not been used long enough to result in a discernible impact. The Afghan government estimates Hib vaccine dose 3 uptake is 98% and PCV13 dose 3 uptake is 89%, but the disparity between official coverage estimates and survey estimates has been greater than 20% in previous years [25]. Further research should investigate the bacterial and viral causes of pneumonia as well as changes in pneumonia incidence once the PCV13 and Hib vaccines have been more widely circulated.

Meningitis had the fewest number of cases of the diseases studied but it had the highest case fatality rate. The case fatality of meningitis that we found (8.8%) was lower than in a study of pneumococcal meningitis in India (36.9%) [26]; but similar to a study from the UK (8%) [27]. We found declines in meningitis throughout the study period. While this decrease in meningitis incidence may again be due to PCV and Hib vaccine use, it is difficult to interpret because meningitis can be caused by a wide variety of different pathogens. The declines seen in meningitis could also be attributable to changes in reporting or diagnostic practices or changes in pathogen composition over time. There was no observed seasonality in meningitis incidence, which may again be due to the presence of multiple causes of meningitis, with cases due to each pathogen peaking in different seasons. In contrast to our study, though, the China study found a large seasonal variation for meningitis [19], and a review of bacterial meningitis in 47 countries found that winter months having the greatest incidence of disease [28]. These differences could be due, in part, to disparities in the etiologic agents of meningitis across different countries. Future studies on the etiology of meningitis (along with pneumonia and diarrhea) can provide more insight into what drives seasonal trends of these diseases, which can be caused by various pathogens.

We found a decline in typhoid cases throughout the study period. Improvements in sanitation could explain the declining rates of typhoid, which has also been endemic in Afghanistan due to breakdowns in sanitary infrastructure from war and economic challenges [10]. While a vaccine against typhoid exists it is generally only recommended to travelers to Afghanistan rather than for routine use in the Afghan population [13]. The decline in typhoid, in the absence of a widespread vaccination program—such as what exists for acute viral hepatitis or meningitis, which also saw declines in incidence, may speak to improvements in sanitation and hygiene. Typhoid cases peaked in the summer, which is in accordance with other studies that have shown increases in typhoid with increases in temperature and rainfall [29]. The China study examining notifiable disease reports also found the greatest typhoid incidence in the summer [19].

The most notable aspect of the measles analysis is the resurgence in measles cases following nationwide supplementary immunization activities (SIAs). Afghanistan has instituted measles SIAs for all children <5 years of age at regular intervals which have taken place in 2009, 2011, and 2015 [25]. Data from 2012 were not available, but we did find significantly lower rates of measles in 2013 and 2014 compared to 2009. However, the resurgence in cases in 2015 points to the difficulty and the necessity of maintaining a cohort that is fully immunized against this highly infectious disease: a study of measles in Tianjin, China, also found that rates of disease bounced back within 2 to 4 years of an SIA [30]. The coverage of the second dose of measles vaccine in Afghanistan is only estimated to be around 60% as of 2015 [25]. Like our study, incidence of measles was highest in May and lowest in October in a study of notifiable disease reports in China from 2005 to 2014 [19].

### Strengths and limitations

This study has several strengths and limitations. It is reasonably comprehensive geographically and reflective of the diversity of Afghanistan by virtue of including sentinel sites from each province in the country but the selection of sentinel sites is not random or systematic. Moreover, different sentinel sites could employ different case definitions for the same disease. Although we controlled for the changing number of sentinel sites over time, the catchment area for each sentinel site differs, and there were periods where no data were collected. These issues could lead to selection biases because the proportion of different types of sentinel sites reporting into DEWS could have changed over time. Because we did not have information on the population of the catchment area or of the types of sentinel sites reporting into DEWS, we could not calculate incidence rates in the general population. Additionally, the population of Afghanistan has grown rapidly in recent years, from 26.5 million in 2009 to 32.5 million in 2015. Therefore, caution should be exercised in interpreting trends across the years. For example, decreases in incidence over time could be from newer sentinel sites having a smaller catchment area and having a lower background incidence of disease.

DEWS includes all suspected cases of disease, and these numbers could vary substantially from figures reported to international agencies. For example, there were 11,531 reports of measles in DEWS in 2015, but only 1,154 cases were reported to the WHO [25]. More widespread laboratory confirmation of cases and investigation of the etiology of pneumonia, diarrhea, and meningitis could better inform disease control efforts and should be included in DEWS reports.

## Conclusions

Despite limitations in Afghanistan’s public health infrastructure, including its surveillance systems, an examination of communicable disease data from DEWS proved useful and informative. We found distinct seasonal patterns for pneumonia, diarrhea, typhoid, and measles, along with declines over time in the occurrence of meningitis, typhoid, and acute viral hepatitis. As sanitary infrastructure improves and vaccinations for pneumococcus, Hib, rotavirus, and other diseases become more accessible to the Afghan population, ongoing analysis of DEWS can help evaluate the effectiveness of disease control efforts in Afghanistan, and can identify targets for future disease control plans.

## Supporting information

S1 Table Cyclical and long-term trends in the incidence of suspected cases of six diseases from Afghanistan’s Disease Early Warning System, 2009–2015. (DOCX)
