# Supplementary material for: Trends of vaccine-preventable diseases in Afghanistan from the Disease Early Warning System, 2009–2015
Source: PLoS One. 2017 Jun 1;12(6):e0178677. doi: 10.1371/journal.pone.0178677 (PMC5453561; doi:10.1371/journal.pone.0178677)
Supplement: S1 Table — (DOCX) [file pone.0178677.s001.docx]

**Supplementary Table 1**. Cyclical and long-term trends in the incidence of suspected cases of six diseases from Afghanistan’s Disease Early Warning System, 2009-2015.

|  | Pneumonia | Diarrhea | Meningitis | Typhoid | Measles | Acute Viral Hepatitis |
| --- | --- | --- | --- | --- | --- | --- |
|  | RR (95% CI) | RR (95% CI) | RR (95% CI) | RR (95% CI) | RR (95% CI) | RR (95% CI) |
| Intercept | 125.59  (112.73, 139.91) | 338.36  (309.95, 369.37) | 3.20  (2.88, 3.56) | 30.81  (28.59, 33.20) | 2.34  (1.82, 3.03) | 3.07  (2.79, 3.38) |
| sin (2π*month/12) | 1.37  (1.27, 1.49) | 0.68  (0.63, 0.73) | 1.01  (0.92, 1.10) | 0.82  (0.77, 0.89) | 1.58  (1.26, 2.00) | 0.94  (0.88, 1.02) |
| cos (2π*month/12) | 1.45  (1.30, 1.61) | 0.55  (0.51, 0.60) | 0.89  (0.82, 0.96) | 0.68  (0.63, 0.73) | 0.58  (0.44, 0.76) | 1.00  (0.90, 1.10) |
| Year (continuous) | 1.00  (0.97, 1.03) | 1.01  (0.98, 1.03) | 0.87  (0.85, 0.90) | 0.87  (0.85, 0.89) | 0.91  (0.84, 0.98) | 0.92  (0.89, 0.94) |
